# The most common diet results in low reproduction in a generalist seabird

**DOI:** 10.1002/ece3.3018

**Published:** 2017-05-20

**Authors:** Susanne van Donk, Kees C. J. Camphuysen, Judy Shamoun‐Baranes, Jaap van der Meer

**Affiliations:** ^1^ Department Coastal SystemsNIOZ Royal Institute for Sea Research and Utrecht UniversityDen BurgTexelThe Netherlands; ^2^ Computational Geo‐EcologyIBEDUniversity of AmsterdamAmsterdamThe Netherlands

**Keywords:** central place forager, compositional analysis, dietary specialization, reproductive consequences, seabird

## Abstract

Dietary specialization has been described across a wide range of taxa in the animal kingdom. Fitness consequences are, however, not well documented. We examined the reproductive consequences of different dietary specializations in the herring gull *Larus argentatus*, an omnivorous seabird, using an extensive dataset which includes breeding and dietary data of 10 successive years. We hypothesized that pairs that focused on prey of higher energetic value would yield higher fledging rates. An alternative hypothesis is that pairs that relied on more resources simultaneously would reproduce better. The novelty of this study is that we used continuous measurements representing dietary composition and degree of specialization rather than restricting our analysis to predefined categories. By relating these two continuous measurements for diet to several proxies for reproductive success, we show clear consequences of dietary choice. Most pairs concentrated on bivalves, a prey type not particularly rich in energy. Pairs feeding on energy‐rich prey (e.g., “domestic refuse and fishery discards”) during chick rearing were found to have a higher reproductive success, supporting the first hypothesis. Pairs that used more resources did not clearly have a higher reproductive success. The majority of the pairs did not switch to energy‐rich prey during chick rearing, despite low breeding outcome. We discuss how trade‐offs between factors such as resource availability, predictability, and the time and energy needed to obtain certain prey species may influence resource selection.

## 1 Introduction

Generalist populations utilize a variety of resources or foraging areas, yet they may consist of individuals that specialize and use only part of the population‐level niche breadth (Araújo, Bolnick, & Layman, 2011; Bolnick et al., 2002). Evidence for individual, dietary specialization is available for many species across a broad range of taxonomic groups (Bolnick et al., 2003; Heinrich, 1976; Masello, Wikelski, Voigt, & Quillfeldt, 2013) and is frequently described (Araújo et al., 2011; Bolnick et al., 2003; Eklöv, Svanbak, Eklo, & Svanba, 2006; Huss, Byström, & Persson, 2008; Layman, Quattrochi, Peyer, & Allgeier, 2007; Roughgarden, 1974). However, the importance of specialization for fitness is not well documented (Ceia et al., 2014; Cucherousset et al., 2011; Woo, Elliott, Davidson, Gaston, & Davoren, 2008). Specializing on a particular resource may for instance have immediate consequences for reproductive success, due to prey‐specific differences in provisioning rates or caloric values when raising young (Golet, Kuletz, Roby, & Irons, 2000; Pierotti & Annett, 1991; Votier, Bearhop, Ratcliff, & Furness, 2004). On the other hand, in a changing world (i.e., natural or human‐induced changes in the food landscape), the predictability of particular resources could decline and generalists might be better off (Dehnhard et al., 2016; Wakefield et al., 2015).

Large gulls, *Laridae,* are a suitable group of species to study fitness consequences of dietary specialization, as they are generalists at the species level, but individual birds have multiple foraging specializations (Ceia & Ramos, 2015). Moreover, their reproductive success and diet are relatively easy to study (Pierotti & Annett, 1987; Watanuki, 1992). Previous studies showed that diet can have a significant impact on the reproductive success of gulls (Hunt, 1972; Pierotti & Annett, 1991; Watanuki, 1992; Weiser & Powell, 2010). More specifically, the proportion of fish in diets was correlated with a higher reproductive success, probably due to the higher energetic value in fish compared to other prey types (Annett & Pierotti, 1999; Bukacińska, Bukaciński, & Spaans, 1996). The (positive or negative) effects of dietary biases toward marine prey, intertidal prey, or anthropogenic waste were partly colony specific (Hunt, 1972; Pierotti & Annett, 1991; Ward, 1973; Watanuki, 1992; Weiser & Powell, 2011). Importantly, most aforementioned studies focused on the effect of diet on population‐level characteristics, for example, by comparing reproductive success between populations with different diets (Hunt, 1972; Ward, 1973; Weiser & Powell, 2010), thereby ignoring dietary specializations of individual pairs. Others investigated consequences of dietary specialization at the individual level using a categorical definition of dietary specialization. They used a threshold value to determine whether an individual is a specialist or generalist (Pierotti & Annett, 1991; Watanuki, 1992), hereby ignoring gradients and potentially losing valuable information.

**Figure 1:**
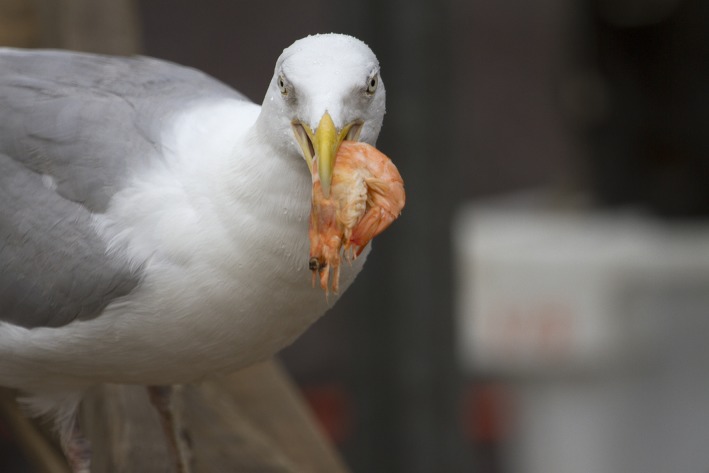
Herring gull with prey. Photograph credit: Maarten van Kleinwee

In this study, we used two continuous measures to study the consequences of dietary specializations of individual breeding pairs in the herring gull *Larus argentatus*, an omnivorous seabird (Figure 1). The first measure focuses on the most prominent prey types in the diet (referred to further as “diet”), whereas the second measure emphasizes the variation within the diet (referred to further as “diversity”). In a long‐term study of the breeding biology and demography of this breeding colony, both dietary preferences and reproductive parameters varied considerably among individual pairs (Camphuysen & Gronert, 2010). However, the consequences of different diets were not explored. The population recovered from a prolonged period of decline, associated with major changes in resources during the 1980s and 1990s (Camphuysen, 2013). During this decline, both breeding success and annual survival were low (Camphuysen & Gronert, 2010; Spaans, de Wit, & van Vlaardingen, 1987). Although the population decline recently came to a halt and numbers stabilized (Boele et al., 2014; Camphuysen, 2013), the birds still experience changes in their food landscape, both within and between seasons such as fluctuations in bivalve quality and availability, changes in fishing fleet densities, and changes in human waste management.

We hypothesized that dietary biases toward prey with higher calorific values would yield a higher reproductive success (Watanuki, 1992). However, in a dynamic food landscape, a specialized lifestyle that relies on few resources might be a vulnerable strategy, especially when energy demands rise during chick care (Huig, Buijs, & Kleyheeg, 2016; Spaans, 1971). An alternative hypothesis, therefore, is that birds with a more diverse diet (generalists) reproduce better (Masello et al., 2013). Using continuous measures for diet and diversity, we use extensive field data to test and compare the two partly contradicting hypotheses. We investigated the egg phase and the chick phase separately, because dietary shifts between phases have been noted in previous studies (Annett & Pierotti, 1989; Murphy, Day, Oakley, & Hoover, 1984; Spaans, 1971) as well as in our own study system (Camphuysen, 2013). We finally discuss breeding consequences of foraging specialization in the context of natural and human‐induced changes in the food landscape.

## 2 Methods

### 2.1 Study area

The study was conducted in a breeding colony on the island of Texel, the Netherlands (53°00′N, 04°43′E), at the crossroads of the western Wadden Sea and the southern North Sea. The main foraging areas include open sea (fish and benthic fauna, including fishery discards), intertidal areas (including mudflats and coastal breakwaters), freshwater ponds, agricultural land, rubbish tips, and cities. Approximately 5,000 herring gulls breed sympatrically with around 10,000 lesser black‐backed gulls, *Larus fuscus,* in the colonies (Camphuysen & Gronert, 2010).

### 2.2 Reproductive measurements

Breeding success within the colony was assessed over 10 consecutive breeding seasons (2006–2015) using study plots that were assumed to represent the colony at large. Only full adults (>4 years old, no immature characteristics within the plumage) were monitored. To monitor breeding success, we marked nests during egg laying (April–May) and marked nests were monitored until hatching (May–June). A subset of these nests were randomly selected and monitored throughout the full breeding season until fledging (July–August). Nest controls were conducted every third day, throughout the breeding season (prospecting through hatching or fledging). When possible, we individually marked birds with a metal and color ring with a unique code (for more details, see Camphuysen & Gronert, 2010).

We used six proxies of reproductive success: timing, egg size, growth rate, fledgling mass, hatching success, and fledging success. To determine timing of a nest, we recorded laying date and subsequently calculated the deviation in days of every individual nest from mean laying date of that particular year. For colony breeders, it is important to synchronize breeding (e.g., not deviating too much from mean laying date) to prevent predation (Hunt & Hunt, 1976; Parsons, 1975; Patterson, 1965). Mean egg volume (egg size) within a clutch was used as a measure of egg quality, as egg size is correlated with chick survival (Krist, 2011). Length (cm) and width (cm) of eggs were measured to the nearest 0.1 mm with Vernier calipers. Egg volume (V, cm^3^) was estimated using the formula of Barth (1968) (*V* = k*L*W ^2^, using *k* = 0.5035 following: Spaans & Spaans, 1975). To be able to determine growth rate, fledgling mass, and fledging success, we enclosed selected nests with high chicken wire fence and marked hatchlings with uniquely numbered aluminum rings (replaced by uniquely numbered stainless steel rings and engraved color rings prior to fledging). During nest visits, we captured chicks from their enclosures. Chicks were measured (head, bill, wing, and tarsus) and weighed with an electronic balance on a quiet place in the colony and returned to the enclosure. Growth rate (g/day) was defined as the slope of the linear regression line between days 5 and 20 of age, as growth rate under optimal conditions is almost linear in this period (Camphuysen, 2013; Oro, Jover, & Ruiz, 1996). Fledging mass was defined as the mass of a chick 40 days after hatching. Hatching success and fledging success were defined as the number of eggs that hatched and the number of chicks surviving until day 40, respectively.

### 2.3 Prey spectrum and prey characteristics

Our study is based on the analysis of regurgitates. Although this method is a widely used method to determine diet, noninvasive, and fairly simple, there is a bias toward hard nondigestible parts so that certain soft tissue prey types that leave no remains at the nest site (such as bread or big bivalves from which gulls do not ingest the shell, like oysters) can be completely overlooked (Weiser & Powell, 2011). Therefore, there is a chance that we missed important prey types. Occasionally, we have nests without prey remains, and these birds probably feed solely on soft prey. These nests were excluded from analysis because of too few food samples.

The dietary analysis was based on regurgitated prey found near the nests of individual pairs. Regurgitates include pellets, boluses obtained when handling adult birds or chicks, and chick feed. Pellet analysis is a widely used method to determine diet in many species, as it is a noninvasive and simple way of collecting large samples over time. Furthermore, it is extensively tested both with captive birds and against other methods (Barrett, 2007). The drawback of this method is that there is a bias toward hard nondigestible parts so that certain soft tissue prey types that leave no remains at the nest site might be underestimated, for instance bread. Boluses and fresh chick feed helped to improve the chances of identifying soft digestible prey.

Chick feed that was still fresh and edible was preferably identified and measured in the field and put back in the enclosure of the corresponding chick. Prey samples were otherwise bagged, labeled, and stored frozen before analysis. Each sample was assigned to the nest‐specific breeding phases (“egg phase” and “chick phase”). Samples were analyzed with a microscope (Olympus SZ51, max. 40× magnification) when needed. Prey types were identified to the lowest possible taxon, measured, and quantified when possible. Sample contamination (blown in or accidently picked up when sampling, e.g., sand, mosses) were excluded from analysis and so were grasses expected to have been ingested to help regurgitate small indigestible remains (e.g., earthworm setae and polychaete jaws). Included plant materials were corn, countryside berries, and plant material originating from domestic refuse.

Only nests that had 10 prey samples or more during the chick rearing phase or egg phase were included in the analysis; this resulted in 141 nests for the egg phase with 16.38 ± 9.12 (mean ± *SD*) prey samples per nest (range 10–70) and 99 nests for the chick phase with 22.11 ± 10.62 (mean ± *SD*) prey samples per nest (range 10–63). After identification, prey types were grouped in 13 main prey categories based on family and/or habitat where prey types could be found: oligochaetes, polychaetes, echinoderms, mammals, freshwater fish, plant matter, terrestrial arthropods (including spiders and woodlice), human waste in the form of domestic refuse, birds (both passerines and nonpasserines, the latter being mostly conspecifics and chicks of the sympatric lesser black‐backed gulls), fishery discards (including marine fish and brown shrimp *Crangon crangon*), intertidal mudflat bivalves, crustaceans, and coastal bivalves (Table 1). We excluded five other dietary components, because of their very low occurrence (<0.1 percent occurrence in total diet): marine gastropods, terrestrial snails, cephalopods, barnacles, and seaweed. One gull regurgitate often contained prey types from several prey categories. Proportions of the 13 prey categories (using frequency of occurrence) were first calculated per regurgitate and subsequently per nest.

**Table 1:** Prey categories and their mean percentages (%; frequency of occurrence) in the diet over all nests used in this study in the egg phase (*n *=* *141) and in the chick phase (*n *=* *107). Each prey category was assigned to one of three main diet components based on a principal component analysis. The main diet components “coastal bivalves,” “domestic refuse and fishery discards,” and “crustaceans and remaining prey categories” were noted as C, D, and R, respectively. Distance, availability, energetic gain, and risk and competition of prey categories were qualitatively assessed (for details regarding the classification, see Appendix S1)

Prey category	Component	Egg phase	Chick phase	Distance	Availability	Energetic gain	Risk and competition
Terrestrial arthropods	R	1.9	2.0	Short	Moderate	Rather high	Rather low
Polychaetes	R	0.3	0.4	Moderate	Rather low	Rather high	Moderate
Oligochaetes	R	0.2	0.5	Short	Moderate	Moderate	Low
Echinoderms	R	0.5	2.2	Moderate	Moderate	Low	Moderate
*Coastal bivalves*	*C*	*69.2*	*43.7*	Short	High	Moderate	Moderate
Mudflat bivalves	R	5.2	0.5	Moderate	Moderate	Moderate	Moderate
Crustaceans	R	6.3	12.1	Short	Rather high	Rather low	Moderate
Freshwater fish	R	1.3	1.1	Long	Moderate	High	Moderate
Fishery discards	D	4.8	16.1	Moderate	Moderate	High	Rather high
Birds	R	4.2	5.7	Short	Rather low	Moderate	High
Mammals	R	1.1	0.5	Moderate	Low	Rather high	Rather high
Domestic refuse	D	3.6	13.8	Long	Rather high	High	High
Plant matter	R	1.4	1.5	Moderate	Moderate	Low	Low

The energy content of different prey categories differs considerably, as do other factors that contribute to the profitability of prey such as handling time, waste materials (digestion), and availability. It is difficult to quantify these proxies exactly, but we assessed the foraging distance, the prey availability, the risks or levels of competition involved while feeding, and the expected energetic gain in categories ranging from favorable (high score) to unfavorable (low score) for each prey category. Traveling distance required to feed ranged from short (<5 km) to long (>50 km) distances, prey availability was classified as “always,” “tidal cycle,” “weather dependent,” “unusually” to “rare opportunities,” and the risks or the competition ranged from low to high. The energetic gain ranged from high to low, incorporating several factors including the potential filling up time (prey size), the energetic value (kJ/g), and the processing time based on the amount of indigestible matter associated with that prey [Appendix S1]. For further details of the food landscape around the study colony, see Camphuysen (2013).

### 2.4 Analyzing the impact of diet

In order to define the most prominent prey types in the population and aggregate prey categories to reduce the number of explanatory variables and facilitate model interpretation, we applied principal component analysis (PCA) to the proportion of prey categories per nest. We used the dietary data from the chick phase in this analysis as the diversity in prey categories is higher in this period. The first two principal components (PC1 and PC2) explained 73 percent of the variance in the data, and thus, we used these principal components to group the prey categories. Loadings of these first two principal components showed us that especially prey categories coastal bivalves, crustaceans, domestic refuse, and fishery discards explained most of the variance in the diet. Loadings of these categories are, respectively, for PC1 [principal component loadings (−0.918; 0.210; 0.220; 0.239) and for PC2 [principal component loadings (−0.020; −0.863; 0.411; 0.274). Prey categories domestic refuse and fishery discards (3rd and 4th values in the loadings) have both a positive and relatively high value for both PC1 as PC2; birds that have domestic refuse in their diet also tend to have more fishery discards in their diet. There seems to be no relation between the other groups (i.e., loadings of coastal bivalves and crustaceans have values that differ greatly from each other as well domestic refuse and fishery discards). Based on these outcomes, we created three “diet components” out of the 13 prey categories: “coastal bivalves” (C), “domestic refuse and fishery discards” (D), and “crustaceans and remaining prey categories” (R). The last group includes crustaceans as well as all the small groups that were of minor influence in explaining the variance in the data (Table 1). These three components (C, D, and R) were used in subsequent analysis.

As we worked with frequencies, all prey categories per nest sum up to one (compositional data). Data that sum up to a constant are not independent of each other. This sum constraint imposes some limitations on the variance and covariance of the data that are non‐normally distributed, invalidating the use of most standard statistical approaches based on multivariate normality. Aitchison (1986) proposed a method that transforms data to remove this sum constraint allowing the use of standard statistical techniques. To transform the data, we recalculated all proportions relative to one randomly chosen proportion (one of the three diet components) and took the log‐transformation of these values. The three diet components in our study are thus described in two coordinates, *z*_1_ and *z*_2_. (Pawlowsky‐Glahn & Egozcue, 2006).

(1) $$z_{1}=\log\left(C/R\right),\;z_{2}=\log\left(D/R\right)$$

The value of one proportion can be deduced from the proportions of the others in compositional data (i.e., food category C must have a value of 1−c, if food categories D and R have together the value of c), and so it does not matter which of the three categories are taken as the denominator. To avoid the problem of calculating log‐ratios over zeros, very small proportions (0.0001) were assumed instead.

The coordinates *z*_1_ and *z*_2_ were used to test our first hypothesis that states that pairs that focus on prey of higher energetic value yield a higher reproductive success. We used linear regression models to test how diet (the predictor) seemingly affected breeding success. During the egg phase, we considered the effect of diet (predictor) on laying date and egg volume (response variables *y*). During the chick phase, diet was considered as the predictor of response variables chick growth and fledging mass. To test whether diet influenced hatching success and fledging success, we used a generalized linear model with a binomial distribution where zero was equal to, respectively, zero hatchlings or zero fledglings and one was equal to one or more hatchlings or fledglings. These models were compared with a null model to test for significance. To visualize and measure the direction of the effect in relation to the three main dietary components, ternary diagrams were created using the ggtern library (Hamilton, 2015), an extension of ggplot in R (Wickham, 2009). Isoclines were included using the coefficients from the linear and generalized models. In a two‐dimensional space where *z*_1_ and *z*_2_ are plotted against each other, the relationship between the response variables and the diet components can be described as follows:

(2) $$y=a_{0}+a_{1}\times z_{1}+a_{2}\times z_{2}$$

when incorporating formulas of *z*_1_ and *z*_2_:

(3) $$y=a_{0}+a_{1}\times \ln\left(C/R\right)+a_{2}\times \ln\left(D/R\right)$$

which is equivalent to the following:

(4) $$y=a_{0}+a_{1}\times \ln\left(C\right)+a_{2}\times \ln\left(D\right)-\left(a_{1}+a_{2}\right)\times \ln\left(R\right)$$

where *z*_1_ and *z*_2_ are the coordinates described in Equation (1), and *y* is the response variable, C, D, and R the three diet components in the ternary diagram, *a*_0_ the intercept of the model, *a*_1_ the coefficient belonging to C, *a*_2_ the coefficient belonging to D, and −(*a*_1_ + *a*_2_) the coefficient belonging to R. The coefficients *a*_1_, *a*_2_, and −(*a*_1_ + *a*_2_) determine the direction of the response. For instance, when *a*_1_ is big and positive and *a*_2_ really small and negative, diet component C has a big positive influence, while diet component D has a small negative influence on the reproductive success. Consequently, component R has a big and negative effect.

Previous studies on gull diet showed a change in diet from the egg phase to the chick phase (Annett & Pierotti, 1989). To evaluate this, we used Hotelling's *T* ^2^ test to compare the means of the prey categories between the egg and chick phases. We used the vectors (*z*_1_ and *z*_2_) that are described above (1) as representation for a pair's diet.

### 2.5 Analyzing diversity

We used the Shannon index for diversity (Roughgarden, 1972) as a continuous measure for diversity to test the second hypothesis that states that pairs that rely on more resources reproduce better.

The Shannon index *H*’ (Shannon and Weaver, 1949) is given by the following equation:

(5) $$H^{\prime }=-\sum \left[p_{i}\times \ln\left(p_{i}\right)\right]$$

where *p*_*i*_ is the proportion of each prey category in the diet of *pair i*. A high value of *H* ^′^ corresponds to a high diversity in the diet (i.e., generalized diet), while a low *H* ^′^ corresponds to low diversity in the diet (i.e., specialized diet).

We used linear regression models to test the effect of diversity (*H* ^′^) during the egg phase on laying date average and egg size and the effect of diversity during chick rearing on average chick growth and average fledging mass. We fit a generalized linear model with a binomial distribution to model the impact of diversity on the probability of hatching or fledging as described above. In line with earlier studies documenting a change in gull diets from the egg phase to the chick phase, we measured whether there was a significant change in diversity between the two breeding phases using a Student's *t*‐test.

## 3 Results

### 3.1 Prey spectrum and prey characteristics

Contribution of the most important prey categories to the overall diet per breeding stage is summarized in Table 1. Coastal bivalves (mostly blue mussels *Mytilus edulis*) are the most prominent prey type in both the egg and the chick phases (Table 1, italic). Dietary composition significantly changed between the two breeding phases [Hotelling's *T* ^2^ test (T.2 = 28.838, *df*1 = 2, *df*2 = 245, *p*‐value <.0001)]. The proportions of crustaceans (mostly common shore crabs *Crangon crangon* and common swimming crab *Liocarcinus holsatus*), domestic refuse (mainly chicken and pork remains and plastic packages of food), and fishery discards (mainly flatfish such as European plaice *Pleuronectes platessa* and common dab *Limanda limanda*, brown shrimp *Crangon crangon* and whiting *Merlangius merlangus*) increased. Note that the energetic content per unit mass of coastal bivalves—while found in the highest proportion in the diet—is lower than human refuse and fishery discards (Table 1).

### 3.2 Diet and reproductive output

Results are summarized in Table 2. The strongest relation that we found was that pairs with more “domestic refuse and fishery discards” in their diet in the chick phase produced chicks with a higher fledging mass (Table 2; large positive *a*_2_). Pairs with more “coastal bivalves” in their diet produced in contrast chicks with low fledging mass (Table 2; large negative *a*_1_). Pairs that feed more on “crustaceans and remaining prey categories” produced chicks of more or less average size (Table 2; relatively small −(*a*_1_ + *a*_2_)). Interestingly, the two other models in the chick phase (growth rate and fledging success) showed the same pattern; pairs that fed more on “domestic refuse and fishery discards” had the best reproductive success (highest growth rate, highest fledging success), while pairs feeding more on “coastal bivalves” had a lower reproductive success (lowest growth rate, lowest fledging success). However, these two models only explained a low amount of the variance (Table 2). We also found that gull pairs feeding more on “coastal bivalves” during the egg phase laid their eggs more closely to the mean laying date (more synchronization) than pairs that fed more on other prey types (Table 2), but this model only explained a low amount of the variance (Table 2).

**Table 2:** Relationships between diet and reproductive output. a1, a2, and –(a1 + a2) are the three coefficients, corresponding to diet components “coastal bivalves,” “domestic refuse and fishery discards,” and “crustaceans and remaining prey categories,” respectively. We used a linear regression model for the upper four response variables, and a generalized linear model with binomial distribution for the last two response variables

Breeding phase	Response variable	Intercept	*a*_1_	*a*_2_	−(*a*_1_ + *a*_2_)	*F*‐value	*r* ^2^	*p*‐value	*df*
Egg	Laying date	−4.619	0.227	0.023	−0.250	4.828	.068	.009**	2, 133
Egg	Egg size	82.052	−0.085	0.053	0.032	0.146	.002	.865	2, 134
Chick	Growth rate	25.362	−1.147	0.996	0.150	4.500	.089	.014*	2, 92
Chick	Fledging mass	787.408	−23.957	33.093	9.136	8.344	.250	.001***	2, 50

Breeding phase	Response variable	Intercept	*a*_1_	*a*_2_	−(*a*_1_ + *a*_2_)	Deviance	*d* ^2^	*p*‐value	*N*
Egg	Hatching success	1.915	0.045	0.066	−0.111	−2.679	0.024	.262	137
Chick	Fledging success	2.102	−0.418	0.236	0.182	−6.108	0.070	.047*	99

* = *p*‐value < .05, ** = *p*‐value < .01, *** = *p*‐value < .001.

**Figure 2:**
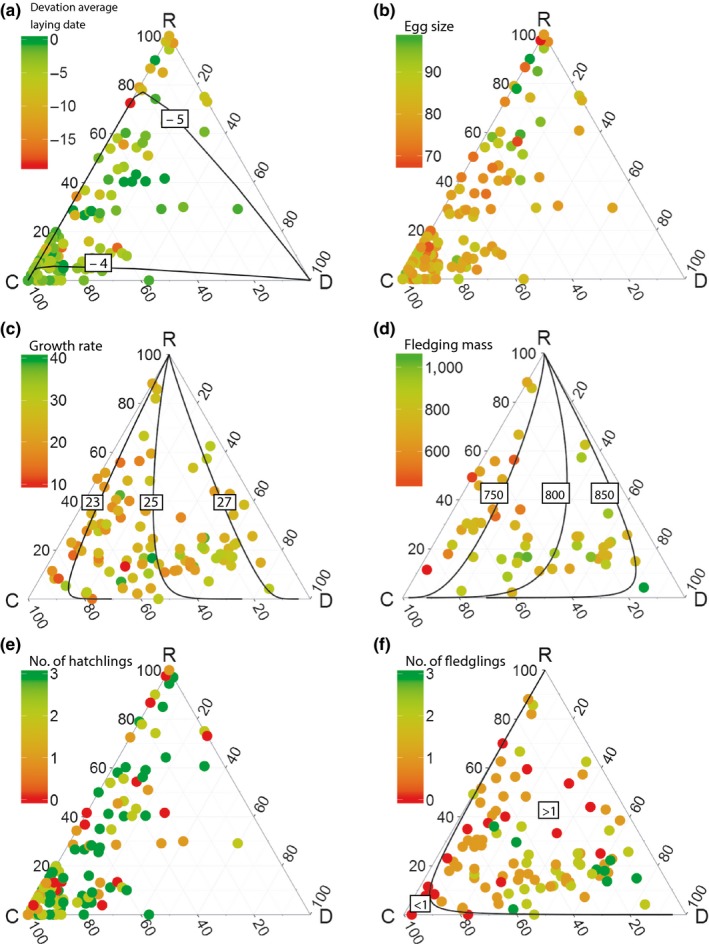
Ternary diagrams that show the relationship between diet and reproductive measurements. The diet components (C, D, and R) in every corner of the diagram correspond to, respectively, “coastal bivalves,” “domestic refuse and fishery discards,” and “crustaceans and remaining prey categories.” When we found a significant relationship between diet and reproductive measurements, expected lines and values are plotted in the diagram. Figure (a), (b), and (e) is results of the egg phase while figure (c), (d), and (f) is results of the chick phase. Every point corresponds to one pair. The color of the point corresponds to the value of the response variable where green corresponds to a high value and red to a low value. Figure (a)–(f) represents diet in relation to (a) the deviation of average laying date in days where a deviation closer to zero corresponds to a more synchronized laying date. (b) Average egg size (cm^3^). (c) Average growth rate (g/day), (d) average fledging mass (grams), (e) hatching success (0 to three eggs hatched), and (f) fledging success (0 to three chicks fledged)

The results are visualized in ternary diagrams in Figure 2 in which also gradients in dietary composition are shown due to our continuous measurements for diet. These diagrams have three corners, one for each of the three primary diet components, referring, respectively, to “coastal bivalves” (C), “domestic refuse and fishery discards” (D), and “crustaceans and remaining prey categories” (R). The location of every point in the ternary diagram represents the diet of a single pair of gulls. When a point is in a corner, for instance near C, the pair fed predominantly on “coastal bivalves.” When a point is in the middle of the diagram, that pair had equal proportions of all prey components in its diet. Each ternary diagram represents another proxy of reproductive success (timing, egg size, growth rate, fledgling mass, hatching success, and fledging success). Significant correlations between diet and a proxy for reproductive success are plotted as isoclines with expected values. The color of points corresponds to the value of the response variables, ranging from red (negative) to green (positive). The strongest results based on model outcomes in Table 2 are shown in Figure 2d: the relationship between dietary components and fledging mass. In this diagram, the transition from red (corresponding to a low fledging mass) close to dietary component “coastal bivalves” to green (a high mass) nearer to “domestic refuse and fishery discards” is most evident.

### 3.3 Diversity and reproductive output

There was no significant relationship between diversity in the diet and breeding output during the egg phase. During the chick phase, pairs with a more diverse diet had a higher reproductive success (higher growth rate and fledging success); however, the relation while statistically significant is very weak (Table 3).

**Table 3:** Relationship between diversity (Shannon index) and reproductive measurements. We used a linear model for the upper four response variables, and a generalized linear model with binomial distribution for the last two response variables

Breeding phase	Response variable	Intercept	*a*_1_	*F*‐value	*r* ^2^	*p*‐value	*df*
Egg	Laying date	−4.222	0.147	0.041	.000	.84	1, 134
Egg	Egg size	80.967	1.163	0.7616	.005	.384	1, 135
Chick	Growth rate	18.889	4.599	5.524	.056	.021^a^	1, 93
Chick	Fledging mass	636.03	104.47	2.592	.048	.114	1, 51

Breeding phase	Response variable	Intercept	*a*_1_	Deviance	*d* ^2^	*p*‐value	*N*
Egg	Hatching success	1.738	0.134	−0.060	0.001	.806	137
Chick	Fledging success	−0.021	1.438	−3.787	0.043	.052	99

a = *p*‐value < .05.

**Figure 3:**
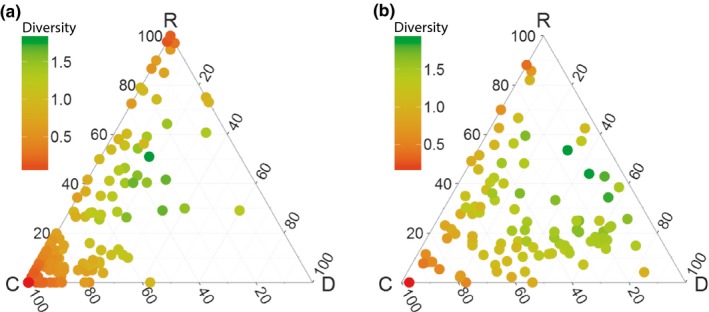
Ternary diagrams that show the relationship between three diet components and the level of diversity of the diet (Shannon index). The diet components (C, D, and R) in every corner of the diagram correspond to, respectively, “coastal bivalves,” “domestic refuse and fishery discards,” and “crustaceans and remaining prey categories.” Every point corresponds to one pair. The color of the point corresponds to the degree of diversity where green corresponds to a high diverse diet, while red corresponds to a low diverse diet. (a) Egg phase, (b) chick phase

Diversity in the diet increased from the egg phase to the chick phase, the average Shannon index increased from 0.67 to 1.23 [*t*‐test (*t *=* *−10.600, *df *= 245, *p*‐value <.0001)]. The change in diversity can also clearly been seen in the ternary diagrams (Figure 3). In the egg phase, most nests are situated in the corners of the ternary diagram with low diversity indicated in red (Figure 3a), while in the chick phase, more nests are in the middle of the ternary diagram reflecting a more diverse diet (Figure 3b).

## 4 Discussion

### 4.1 Main findings

In this study, we investigated two partly contradicting hypotheses using a continuous measure for diet and diversity. The advantage of using continuous scales is that gradients in diet composition or diversity can be revealed. The first hypothesis assumed that pairs with a diet of higher energetic value yield in higher reproductive success. The second assumed a higher reproductive success for pairs with a diverse diet (generalists), as the prey landscape is dynamic in space and time. We found that pairs with a high percentage of energy‐rich prey in their diet (e.g., “domestic refuse and fishery discards”) have a higher reproductive success, mainly in terms of a higher fledging weight of the chicks, than individuals that continued foraging on energy‐poor prey such as mussels, thus supporting the first hypothesis. We did not find strong evidence for the second hypothesis. Higher diversity in the diet during the chick phase increased the probability of fledging chicks, but the relationship explained only a minor fraction of the total variation.

### 4.2 Diet

Pairs that fed on energy‐rich prey had heavier and more fledglings. However, there were also many pairs feeding mainly on energetically poorer prey (coastal bivalves, mainly mussels) in both breeding phases. Those pairs had smaller and fewer fledglings. To understand why numerous pairs do not switch to energy‐rich prey, it may be important to examine the resource characteristics and the costs involved to obtain and process particular prey types (Table 1; details and explanations in Appendix S1). Coastal bivalves, for example, have low intake benefits (low energetics with a lot of indigestible remains: Sibly & McCleery, 1983), but are predictable at short distances around the colony. Handling costs and risks involved are also low (Appendix S1). Energetically much richer prey types such as fishery discards are less predictable, require greater traveling distances, and have high handling costs (Appendix S1). Herring gulls obtain fishery discards exclusively from near‐shore fleets of beam trawlers (targeting shrimp, with small fish as bycatch) within the western Wadden Sea and North Sea coastal zone (Berghahn & Rösner, 1992; Camphuysen, 1995). Anthropogenic (domestic) refuse is, like fishery discards, also not predictable compared to coastal bivalves. Most refuse is taken from cities or from a rather distant landfill area. Domestic refuse is probably also taken from recreational areas, mostly during public holidays and nice weather. Foraging on coastal bivalves might thus be a more predictable strategy and less energy‐consuming. Such a “slower” pace of life may result in a higher survival, which may compensate for the lower annual reproductive success (Réale et al., 2010). Strategies could have equal payoffs in terms of lifetime reproductive success, and the population might be made up by several coexisting and successful strategies. To investigate this hypothesis, we should study survival of individuals with different strategies in addition to reproductive success. Variation in survival between different specializations has been reported in birds (Durell, Goss‐Custard, Caldow, Malcolm, & Osborn, 2001).

The density of competitors could also explain the variation in strategies in the population (Bolnick, 2004; Svanbäck & Persson, 2009). When many individuals try to get to the most favorable prey types, competition increases. Individuals that forage on less favorable prey types have fewer competitors to share these resources with and so their overall fitness might be relatively high (Bolnick, 2004). We should study interference competition of different prey types in our study to find out whether the density of competitors plays a role. Van de Pol and colleagues (2010) did test whether there was selective pressure on density in an extensive, long‐term study on oystercatchers. They could not find evidence for this hypothesis although population size might have been too small for competition to take place. They did find strong fluctuations in survival between different strategies over years, suggesting that variation in strategies persists in the population due to changing conditions in the environment.

### 4.3 Diversity

We did not find strong evidence that higher diversity in the diet (more generalist diet) results in higher reproductive success. Higher diversity in the diet during the chick phase increased the probability of fledging chicks, but the relationship was statistically weak. However, we did report a strong significant increase in diversity in the diet from the egg phase to the chick phase. During the egg phase, diversity in the diet is quite low (more specialization), while during the chick phase, the diet becomes more diverse. A higher diversity in prey items during the chick phase was primarily caused by a higher percentage of crustaceans, fishery discards, and domestic refuse. This suggests that more diversity in the diet during chick phase indirectly results in a higher reproductive success, but only when the diet incorporates energy‐rich prey such as domestic refuse and fishery discards and not by adding for instance crustaceans. Diet composition also clearly changed between the two breeding phases (Table 1). An increase in the proportion of fish and domestic refuse was reported in the diet during the chick phase. This seems to be a switch in diet and not a change in the availability as fishing vessels are active in the same amount all year long (Camphuysen, 2013, p. 55), and there are also no indications that domestic refuse changes dramatically over the breeding season. Diet switches over the course of the breeding season have been frequently reported for seabirds (Murphy et al., 1984; Ward, 1973). In some studies, birds switched to a diet with more fish as soon as the chicks were hatched (Annett & Pierotti, 1989; Spaans, 1971), probably caused by an increase in the energetic demands or by special nutritional requirements of the chicks (Annett & Pierotti, 1989).

### 4.4 Other variables

Age (Elliott, Duffe, Lee, Mineau, & John, 2006; Monaghan, 1980), individual characteristics (i.e., “personality,” morphology) (Patrick & Weimerskirch, 2014; Sol et al., 2005), and experience (Limmer & Becker, 2009; Werner, Mittelbach, & Hall, 1981) can influence both reproductive success and foraging strategies. Life‐history theory predicts for instance that effort toward reproduction increases with age in long‐lived organisms (Paitz, Harms, Bowden, & Janzen, 2007). We may be able to evaluate some of these factors in years to come when more individuals have been monitored for a number of years and their diet, morphology, and age assessed. Sex could also influence foraging strategies, but we were unable to distinguish between diets of males and females within a pair. Differences in foraging strategies between sexes have been reported in lesser black‐backed gulls using GPS loggers, however (Camphuysen, Shamoun‐Baranes, Van Loon, & Bouten, 2015), suggesting in that case that males specialized more strongly on marine habitats and fish prey and females on a diversity of habitats closer to the shore and on land (suggesting a greater diversity of prey). Sex differences (associated with sexual dimorphism) in habitat use and diet were also reported in raptors and snakes (Andersson & Norberg, 1981; Shine, Harlow, & Keogh, 1998).

Our study is based on the analysis of regurgitates. Although this method is a widely used and excepted method to determine diet, noninvasive, and fairly simple, there is a bias toward hard nondigestible parts so that certain soft tissue prey types that leave no remains at the nest site (such as bread or big bivalves from which gulls do not ingest the shell, like oysters) can be completely overlooked (Weiser & Powell, 2011). Therefore, there is a chance that we missed important prey types. Occasionally, we have nests without prey remains and these birds probably feed solely on soft prey. These nests were excluded from analysis because of too few food samples.

## 5 Conclusion and future directions

Our study shows that not all pairs have a diet that would lead to a higher fledging success (energy‐rich prey including discards and/or human waste). Many pairs fed mainly on coastal bivalves even though this diet was related to a lower reproductive output. Differences in diet between individuals could also be reflected in differences in survival. Successful breeding birds—pairs that feed on human waste and fishery discards—might actually have a lower survival and lifespan as a result of higher costs involved with successful reproduction (Blomberg, Sedinger, Nonne, & Atamian, 2013; Creighton, Heflin, & Belk, 2009; Smith & Blumstein, 2008). Individuals that relied on lower energy, predictable prey throughout the breeding season could, by contrast, increase their survival by lowering foraging costs and potentially increasing lifetime reproductive success. Tracking individuals with GPS loggers with integrated accelerometers to estimate time energy budgets of different foraging specializations and studying survival are likely to provide more insights into trade‐offs associated with different foraging specializations in future.

## Conflict of interest

None declared.

## Data accessibility

Raw data files used in the analysis are available on 4TU. Centre for Research Data; https://doi.org/10.4121/uuid:386de9a1-e9a2-4c48-83f1-40f49e91f470.
